# Reduced knee laxity and failure rate following anterior cruciate ligament reconstruction compared with repair for acute tears: a meta-analysis

**DOI:** 10.1186/s10195-023-00688-5

**Published:** 2023-02-20

**Authors:** Filippo Migliorini, Gianluca Vecchio, Jörg Eschweiler, Sarah-Marie Schneider, Frank Hildebrand, Nicola Maffulli

**Affiliations:** 1 Department of Orthopaedic, Trauma, and Reconstructive Surgery, RWTH University Hospital, Pauwelsstraße 31, 52074 Aachen, Germany; 2 Department of Orthopaedic and Trauma Surgery, Eifelklinik St. Brigida, 52152 Simmerath, Germany; 3 Department of Medicine, Surgery and Dentistry, University of Salerno, Via S. Allende, 84081 Baronissi, SA Italy; 4 School of Pharmacy and Bioengineering, Faculty of Medicine, Keele University, Thornburrow Drive, Stoke On Trent, England; 5 Barts and the London School of Medicine and Dentistry, Centre for Sports and Exercise Medicine, Mile End Hospital, Queen Mary University of London, 275 Bancroft Road, London, E1 4DG England

**Keywords:** Knee, ACL reconstruction, Conservative, Treatment

## Abstract

**Background:**

Following anterior cruciate ligament (ACL) tears, both repair and reconstruction may be performed to restore joint biomechanics and proprioception. The present study compared joint laxity, patient-reported outcome measures (PROMs), and rate of failure following primary repair versus reconstruction for ACL ruptures.

**Methods:**

This meta-analysis followed the Preferred Reporting Items for Systematic Reviews and Meta-Analyses guidelines. Pubmed, Google scholar, Embase, and Web of Science were accessed in September 2022. All the clinical investigations comparing repair versus reconstruction for primary ACL tears were accessed. Studies reporting data on multiple ligament injuries settings were not eligible.

**Results:**

Data from eight articles (708 procedures) were collected. The mean length of the follow-up was 67.3 ± 119.4 months. The mean age of the patients was 27.1 ± 5.7 years. Thirty-six percent (255 of 708 patients) were women. The mean body mass index (BMI) was 24.3 ± 1.1 kg/m^2^. The mean time span from injury to surgery was 36.2 ± 32.3 months. There was comparability at baseline with regards to instrumental laxity, Lachman test, International Knee Document Committee (IKDC), and Tegner Scale (*P* > 0.1). Similarity between ACL reconstruction and repair was found in IKDC (*P* = 0.2) and visual analog scale (VAS) satisfaction (*P* = 0.7). The repair group demonstrated greater mean laxity (*P* = 0.0005) and greater rate of failure (*P* = 0.004).

**Conclusion:**

ACL reconstruction may yield greater joint stability and lower rate of failure compared with surgical repair. Similarity was found in PROMs.

**Level of evidence:**

III

## Introduction

Anterior cruciate ligament (ACL) tears are common [1]. The incidence of acute ACL injuries has been estimated to be up to 78 per 100,000 individuals in the general population [2, 3]. ACL tears are relatively common in active and young individuals [4–7]. The management of ACL tears aims to restore knee joint kinematics, preventing instability, and enhancing the activity level of the patients [8–10]. Both arthroscopic repair and reconstruction are viable strategies for ACL tears. Primary repair of proximal ACL tears was first described in 1903 [11]. The past decade has seen a growing interest in ACL repair [12–16]. ACL repair avoids tunnel drilling and graft harvesting, thus reducing morbidity and allowing a fast recovery [17–19]. Furthermore, this procedure is believed to better preserve proprioception [20, 21]. However, ACL repair is advocated only in acute settings, within 6 weeks from the injury [22]. Arthroscopic ACL reconstruction using an autograft has been widely performed [23, 24]. Hamstrings, quadriceps, or patellar tendon autografts are commonly used [25, 26]. There are still concerns whether ACL repair produces results comparable to reconstruction [22, 27–31]. To the best of our knowledge, no previous meta-analysis that summarizes the evidence of repair versus reconstruction are available. The purpose of the present meta-analysis was to compare primary reconstruction versus ACL repair for ACL tears, in laxity, patient-reported outcome measures (PROMs), and rate of failure.

## Material and methods

### Eligibility criteria

All the clinical investigations comparing arthroscopic reconstruction versus ACL repair for acute ACL tears were accessed. Articles in English, German, Italian, French, and Spanish, according to the authors language capabilities, were considered. Studies with level I–III of evidence, according to the Oxford Centre of Evidence-Based Medicine [32–34], were eligible. Studies that performed ACL reconstruction/repair in a multiple ligament damage setting were not eligible. Only studies that performed primary ACL surgery were considered. Expert opinions, technical note, reviews, letters, comments, and editorials were not eligible. Cadaveric, animals, and biomechanics studies were not considered. Studies that investigated multi-ligament injury or revision settings were not considered. Only studies reporting a minimum of 6 months follow-up were eligible. Only articles reporting quantitative data under the outcomes of interest were considered for inclusion.

### Search strategy

This meta-analysis followed the Preferred Reporting Items for Systematic Reviews and Meta-Analyses (PRISMA) guidelines [35–38]. The PICO(TS) algorithm was preliminary pointed out:

- P (population): ACL tears;
- I (intervention): ACL repair;
- C (comparison): ACL reconstruction;
- O (outcomes): laxity, PROMs, failures.
- T (timing):  ≥ 6 months.
- S (study type): clinical investigation

### Data source

Two authors independently (S.M.S. and F.M.) performed the literature search accessing the following: PubMed, Google Scholar, Embase, and Web of Science databases in September 2022. The following keywords were used in combination: knee, anterior cruciate ligament, ACL, damage, injury, tear, rupture, management, treatment, arthroscopy, surgery, reconstruction, repair, patient reported outcome measures, PROMs, laxity, stability, instability, function, quality of life, failures. The same authors independently analyzed resulted titles and abstracts. If the abstract matched the topic, the article full text was accessed. The bibliographies of the full-text articles were also screened. Disagreements between the authors were solved by a third author (N.M.).

### Data extraction

Two authors (S.M.S. and F.M.) performed data extraction in a separate fashion. Author, year of publication, journal, and study design were collected. Data concerning the demographic of the included patients at baseline were retrieved: age, gender, body mass index (BMI), time elapsed from injury to surgery, and length of the follow-up. Data on instrumental laxity and the International Knee Document Committee (IKDC) were collected at baseline to assess between groups comparability. Data on instrumental laxity, IKDC [39], and visual analog scale (VAS) [40] were collected at last follow-up. The rate of failure at last follow-up was also retrieved. The instrumental laxity was evaluated using the KT-1000 and KT-2000 (MEDmetric Corp, San Diego, California) arthrometers. Both these devices applied a force of 134 N on the proximal tibia over the femur condyles directed anteriorly and evaluated the joint displacement in mm.

### Methodology quality assessment

The methodological quality assessment was made using the risk of bias summary graph of the Review Manager Software version 5.3 (The Nordic Cochrane Collaboration, Copenhagen). The following risk of bias were evaluated: selection, detection, reporting, attrition, and other source of bias.

### Statistical analysis

The statistical analyses were performed by two authors (S.M.S. and F.M.). For descriptive statistics, SPSS software version 25 was used. The mean and standard deviation (SD) were calculated. To assess baseline comparability of the continuous variables, the Student’s *t*-test was performed, with values of *P* > 0.1 considered satisfactory. Review Manager Software version 5.3 (The Nordic Cochrane Collaboration, Copenhagen) was used for the meta-analyses. The inverse variance was adopted for continuous variables, with mean difference (MD) effect measure. Dichotomic data were evaluated through a Mantel–Haenszel analysis, with odds ratio (OR) effect measure. A fixed model effect was used in all the comparisons. Heterogeneity was assessed through the $\chi$ ^2^ test and Higgins-I^2^ test. If $\chi$ ^2^ < 0.05 and *I*^2^ test > 50%, high level of heterogeneity was detected and a random model effect was adopted. Values of *P* < 0.05 were considered statistically significant. A funnel plot was performed to assess the overall risk of publication bias. Egger’s linear regression was performed using STATA MP Software version 16 (StataCorp, College Station, USA) to assess plot asymmetry. Values of *P*_Egger_ < 0.05 indicated statistically significant asymmetry.

## Results

### Search result

**Fig. 1:**
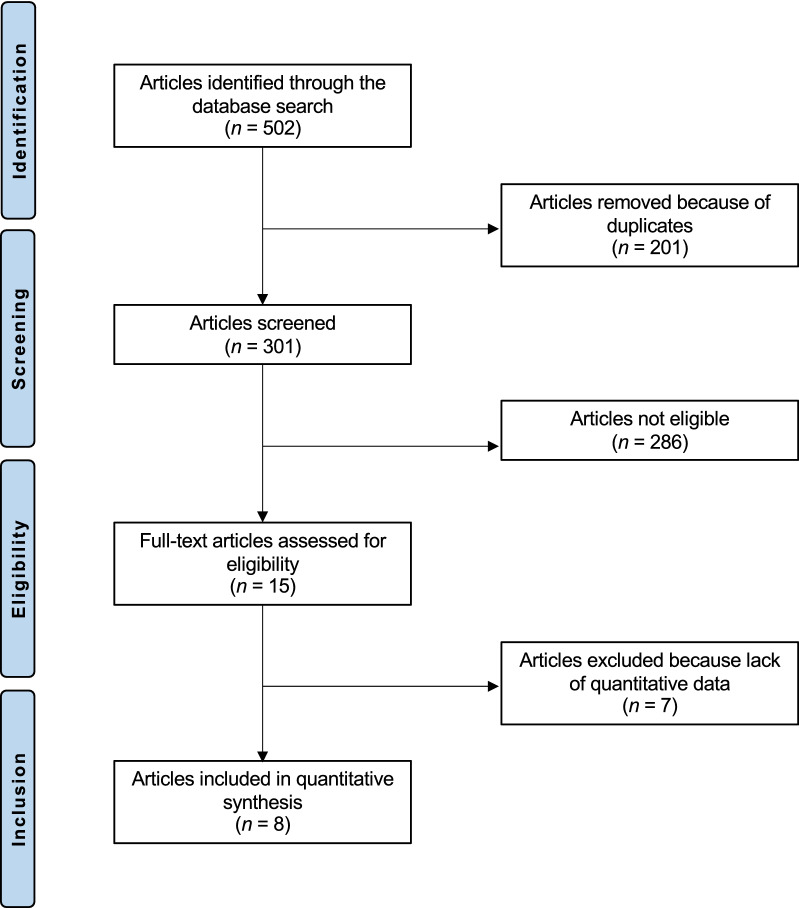
Flow chart of the literature search

The initial literature search resulted in 502 articles. Of these, 201 were excluded because of duplication. A further 286 articles were not eligible as they did not satisfy the eligibility criteria: incorrect study type (*N* = 104), not matching topic (*N* = 163), concerning revision setting (*N* = 6), performing combined intervention (*N* = 13). A further seven articles did not report quantitative data under the outcomes of interest, and therefore were not included in the present study. This left eight studies for the present investigation (Fig. 1): five randomized controlled trials [27–30, 41], one prospective [22], and two retrospective clinical investigations [17, 42].

### Methodological quality assessment

**Fig. 2:**
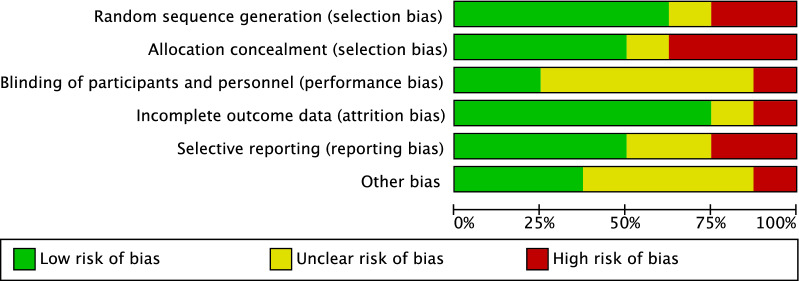
Methodological quality assessment

Given the prospective nature of 75% (six out of eight) of the studies, along with 63% (five out of eight) of studies that performed randomized allocation, the risk of selection bias was low–moderate. Assessor blinding was seldom performed and often biased, leading to a moderate risk of performance bias. The risk of attrition and reporting biases was low–moderate. The risk of other biases was moderate. Therefore, the methodological quality assessment detected a low–moderate risk of bias (Fig. 2).

### Risk of publication bias

**Fig. 3:**
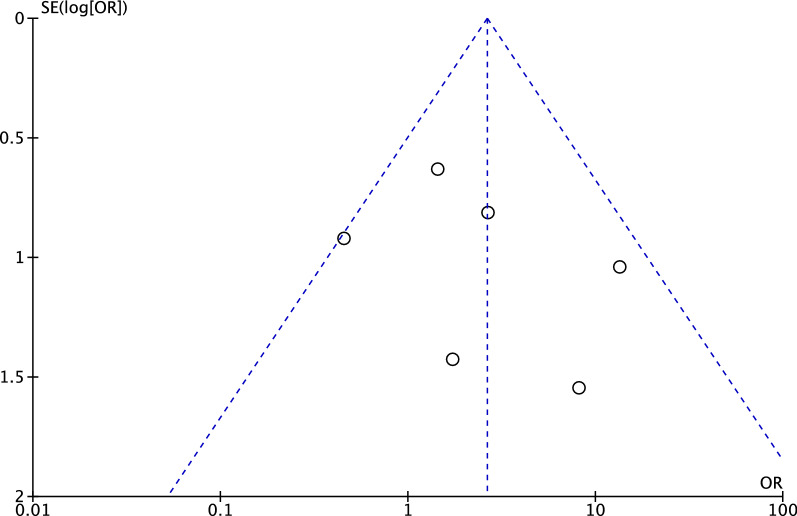
Funnel plot of the most reported outcome (failure)

To assess the risk of publication bias, the funnel plot of the most commonly reported outcome (failure) was investigated (Fig. 3). The Egger’s test was not significant (*P* = 0.5), indicating no statistically significant asymmetry. Concluding, the plot revealed low risk of publication bias.

### Patient demographics

Data from 708 procedures were collected. The mean length of the follow-up was 67.3 ± 119.4 months. The mean age of the patients was 27.1 ± 5.7 years. Thirty-six percent (255 of 708 patients) were women. The mean BMI was 24.3 ± 1.1 kg/m^2^. The mean time from injury to surgery was 36.2 ± 32.3 months. Study generalities and surgical techniques are presented in Tables 1 and 2, respectively. There was comparability at baseline with regard to patient demographics, length of time to surgery and follow-up, and IKDC (Table 3).

**Table 1:** Study generalities and patient demographics

Author	Journal	Design	Follow-up (months)	Treatment	Patients (*n*)	Mean age	Female (%)
Achtnich et al. 2016 [22]	*Arthroscopy*	Prospective	28	Repair	20	30	N/A
				Reconstruction	20	33.6	N/A
Hoogeslag et al. 2019 [27]	*Am J Sports Med*	RCT	24	Repair	24	21	21
				Reconstruction	24	22	25
Kosters et al. 2020 [28]	*Am J Sports Med*	RCT	24	Repair	43	28.7	42
				Reconstruction	42	27.6	26
Murray et al. 2020 [29]	*Am J Sports Med*	RCT	24	Repair	65	17	37
				Reconstruction	35	17	19
Schliemann et al. 2017 [30]	*Knee Surg Sports Traumatol Arthrosc*	RCT	12	Repair	30	28.2	50
				Reconstruction	30	29.1	27
Sporsheim et al. 2019 [41]	*J Bone Joint Surg Am*	RCT	360	Repair	99	N/A	44
				Reconstruction	51	N/A	44
Vanderlist et al. 2017 [17]	*The Knee*	Retrospective	6	Repair	52	33	42
				Reconstruction	90	29	39
Vermeijden et al. 2020 [42]	*Arthroscopy*	Retrospective	60	Repair	49	34.4	49
				Reconstruction	34	29.4	41

RCT: randomized control trials

**Table 2:** Surgical techniques

Author	Journal	Technique	Surgical procedure	Procedures number
Achtnich et al. 2016 [22]	*Arthroscopy*	Repair	Arthroscopic suture anchor repair	20
		Reconstruction	4SHT	20
Hoogeslag et al. 2019 [27]	*Am J Sports Med*	Repair	DIS	24
		Reconstruction	4SHT	24
Kosters et al. 2020 [28]	*Am J Sports Med*	Repair	DIS	43
		Reconstruction	4SHT	42
Murray et al. 2020 [29]	*Am J Sports Med*	Repair	BEAR	65
		Reconstruction	4SHT	33
			BPTB	2
Schliemann et al. 2017 [30]	*Knee Surg Sports Traumatol Arthrosc*	Repair	DIS	30
		Reconstruction	4SHT	30
Sporsheim et al. 2019 [41]	*J Bone Joint Surg Am*	Repair	Open ACL repair	39
			Open ACL repair with LAD	39
		Reconstruction	BPTB	35
Vanderlist et al. 2017 [17]	*The Knee*	Repair	Arthroscopic suture anchor repair	52
		Reconstruction	4SAT	49
			BPTB	38
			4SHT	3
Vermeijden et al. 2020 [42]	*Arthroscopy*	Repair	Arthroscopic suture anchor repair	49
		Reconstruction	4SAT	14
			BPTB	9
			4SHT	7

4SHT: four-strand hamstring tendon; DIS: dynamic intraligamentary stabilization; BEAR: bridge-enhanced ACL repair; BPTB: bone–patellar tendon–bone; LAD: ligament augmentation device; 4SAT: four-strand allograft tendon

### Meta-analyses

**Fig. 4:**
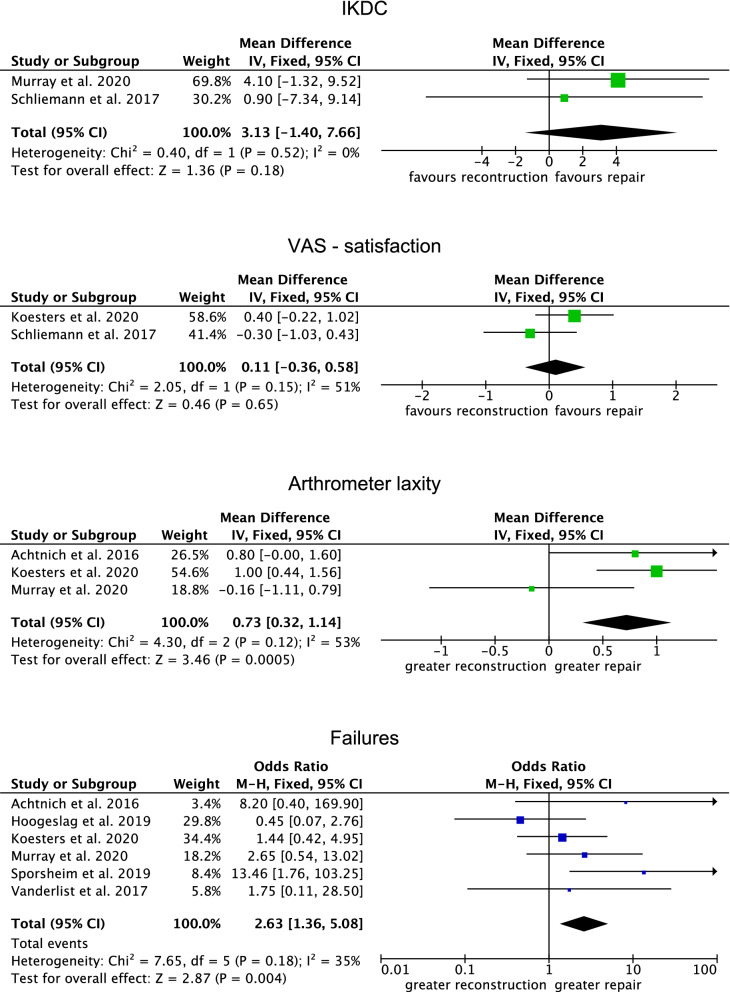
Forest plots

Similarity between ACL reconstruction and repair was found in IKDC (*P* = 0.2) and VAS satisfaction (*P* = 0.7). The repair group demonstrated greater mean laxity (MD 0.73; 95% CI 0.32–1.14; *P* = 0.0005) and greater rate of failure (OR 2.63; 95% CI 1.36–5.08; *P* = 0.004). Further details of these results are given in Fig. 4.

**Table 3:** Comparability of the baseline between the two groups (mean and standard deviation)

Endpoints	Reconstruction (*N* = 326)	Repair (*N* = 382)	*P*-value
Follow-up (months)	67.3 ± 119.4	99.8 ± 148.3	0.6
Age (years)	26.8 ± 5.5	27.5 ± 6.3	0.8
BMI (kg/m^2^)	24.6 ± 1.1	23.9 ± 1.1	0.3
Women (%)	31.5 ± 9.6	41.1 ± 9.2	0.1
Time from injury to surgery (days)	100.8 ± 154.0	27.1 ± 14.8	0.3
IKDC (0–100)	56.7 ± 10.0	60.7 ± 11.2	0.7

No statistically significant difference was found in the endpoints of interest, indicating good between-groups comparability

BMI: body mass index; IKDC: International Knee Document Committee

## Discussion

According to the main findings of the present meta-analysis, ACL reconstruction yielded greater joint stability and lower rate of failure compared with surgical repair. Similarity was found in PROMs.

In a recent meta-analysis involving 1263 patients, Biau et al. concluded that only 40% of patients return to their previous activity levels after ACL reconstruction [43]. Given these findings, to optimize the clinical results of surgery for ACL ruptures, a renewed interest has emerged on ACL suture repair. Cruciate ligament repair may be considered the first attempt to restore the integrity of natural tissues [44]. Strand et al. [45] found a failure rate of 27% after open suture repair at a minimum of 10 years follow-up, and concluded that open ACL repair should no longer be recommended. Vanderlist et al. [17] found that patients who underwent ACL repair demonstrated earlier return to full range of motion compared with patients following arthroscopic reconstruction. Furthermore, the repair procedure required significantly shorter surgical times than reconstruction surgery [17]. A previous systematic review [46] investigated the clinical outcomes of primary ACL repair, recommending the dynamic intraligamentary stabilization (DIS) technique for optimal outcomes.

The ACL has two components, the anteromedial bundle and the posterolateral bundle [47]. In vitro, the anteromedial bundle has a certain tension [48]. When the knee joint is flexed between 20° and 90°, the tension will increase in the anteromedial bundle, while, when the knee extends, the tensile force on posterolateral bundle increases [48, 49]. The ACL also functions as a major secondary constraint for internal rotation, especially when the joint is close to full extension [50]. In addition, the ACL exerts a slight secondary restraint effect on external rotation and varus–valgus angle, especially under load [51, 52]. The ACL contains several mechanoreceptors involved in proprioception [53]. Static and dynamic information regarding the joint, especially regarding position awareness, detection of movement, and acceleration, are collected by the knee proprioceptors, allowing, in addition, a closed-loop nervous activity [20]. These features are strictly involved in joint movement control, avoiding aberrant motion, which may lead to further injuries of ligaments and menisci [54]. Therefore, the ACL plays a major role to preserve knee stability during motion, especially in sport activities when complex movements are required [55].

The basic principle of ACL biology and healing after graft implantation is an inflammatory response [56]. Neutrophils and macrophages progressively repopulate the tendon graft and contribute to the formation of a fibrous scar tissue interface between the graft and bone tunnel through the action of cytokines and growth factors [57]. After 6 weeks, the graft is completely covered by a vascular synovial envelope, and at 6 months the intrinsic vasculature of the intra-articular portion graft is complete [58, 59]. The remodeling phase of the intra-articular portion of the graft tissue, ‘‘ligamentization,’’ is characterized by the replacement of collagen fibrils, which gradually assume the histological properties of the native ACL [60–62]. At 8 months, the percentage of type III collagen, glycosaminoglycan, and cross-linking collagen is comparable to those in normal ACL [60]. The number of fibroblasts grows until 1 year following the operation; the number of fibroblasts and blood vessels then decrease, and at 3 years the metabolic activity ceases [63]. The type of graft can affect the healing time. In the bone tunnel, the bone plug showed complete healing at 8 weeks, while healing takes 12 weeks when tendon-to-bone is desired [64]. The biological healing time provides evidence that the safe return to sport after ACL reconstruction should preferably be recommended from 6 to 9 months postoperatively.

ACL reconstruction can be performed with several techniques and grafts [65]. Given the biomechanical properties and the low-harvest morbidity, hamstring tendon grafts are widely used in ACL reconstruction [66]. Despite the possibility that rotational instability might occur, the use of a single-bundle bone–patellar tendon–bone autograft demonstrates a low failure rate and fast graft incorporation [67]. Allografts are a valid option to avoid graft harvest morbidity; however, given the higher costs, risk of disease transmission, and immune reactions, their use remains limited [68]. Three studies [27, 28, 30] augmented the ACL suture repair with the dynamic intraligamentary stabilization (DIS) technique [69]. Sporsheim et al. [41] employed the synthetic ligament augmentation device (LAD) for the repair [70]. Other authors [17, 22, 42] performed an arthroscopic primary ACL repair with suture anchor fixation of the anteromedial and posterolateral bundle [71]. Murray et al. [29] used a bridge-enhanced ACL repair (BEAR) technique [72, 73]. Biomechanically, ACL repair achieved similar anterior tibial translation to noninjured knees at 30° and 90° compared with ACL-reconstructed knees [74]. However, this difference was less than a millimeter, which may be considered as not clinically relevant [74]. In knees with insufficient or ruptured ACL, the amount of tibial anterior translation over the femur is fourfold greater than in a healthy joint [75].

This study has several limitations. The small number of included studies and related sample size represent important limitations. As a consequence of the limited quantitative data available for inclusion, no analysis regarding the various repair techniques could be performed. Given the limited quantitative data for analysis, ACL tears location (proximal or midsubstance) was not considered for analysis. Among the included studies, three included patients with only proximal tears [17, 22, 42], four studies included patients with both proximal and midsubstance tears [27, 28, 30, 41], and one study included patients with midsubstance tears only [29]. Given their greater vascularization, proximal ACL tears have greater healing potential compared with midsubstance ruptures [76–78]. Most authors performed ACL repair and reconstruction in an arthroscopic fashion; only Strand et al. [45] reported data on open suture repair. Although some studies reported a potentially positive effect on proprioception, given the lack of quantitative data and reliable methods to objectivate this, it was not possible to properly investigate. Given the lack of quantitative data and/or missing information, it was not possible to investigate and assess whether general laxity might influence the outcome. Given the lack of quantitative data in the literature, the analyses were conducted regardless of whether single or double bundle reconstruction had been performed, thus representing another potential limitation. Given the lack of quantitative data, it was not possible to analyze the different autografts and/ or surgical techniques separately. The relatively short-term duration of the follow-up may also represent another limitation, and further clinical trials providing long-term follow-up are strongly recommended to establish seldom complications and accurate failure rate. Given these limitations, data from the present study must be interpreted with caution.

## Conclusion

Arthroscopic reconstruction should be recommended for primary ACL tears. Though similarities were found in PROMs between the techniques, ACL reconstruction demonstrated lower joint laxity and rate of failure compared with the repair technique.

## Data Availability

The datasets generated during and/or analysed during the current study are available throughout the manuscript.

## Abbreviations

ACL: Anterior cruciate ligament
PROMs: Patient-reported outcome measures
IKDC: International Knee Document Committee
VAS: Visual analog scale

## References

[1] Gianotti SM, Marshall SW, Hume PA (2009). Incidence of anterior cruciate ligament injury and other knee ligament injuries: a national population-based study. J Sci Med Sport.

[2] Frobell RB, Lohmander LS, Roos HP (2007). Acute rotational trauma to the knee: poor agreement between clinical assessment and magnetic resonance imaging findings. Scand J Med Sci Sports.

[3] Sanders TL, Maradit Kremers H, Bryan AJ (2016). Incidence of anterior cruciate ligament tears and reconstruction: a 21-year population-based study. Am J Sports Med.

[4] Dai B, Herman D, Liu H (2012). Prevention of ACL injury, part I: injury characteristics, risk factors, and loading mechanism. Res Sports Med.

[5] Buller LT, Best MJ, Baraga MG (2015). Trends in anterior cruciate ligament reconstruction in the United States. Orthop J Sports Med.

[6] Dodwell ER, Lamont LE, Green DW (2014). 20 years of pediatric anterior cruciate ligament reconstruction in New York State. Am J Sports Med.

[7] Mall NA, Chalmers PN, Moric M (2014). Incidence and trends of anterior cruciate ligament reconstruction in the United States. Am J Sports Med.

[8] Lohmander LS, Englund PM, Dahl LL (2007). The long-term consequence of anterior cruciate ligament and meniscus injuries: osteoarthritis. Am J Sports Med.

[9] Claes S, Hermie L, Verdonk R (2013). Is osteoarthritis an inevitable consequence of anterior cruciate ligament reconstruction? A meta-analysis. Knee Surg Sports Traumatol Arthrosc.

[10] Boer BC, Hoogeslag RAG, Brouwer RW (2018). Self-reported functional recovery after reconstruction versus repair in acute anterior cruciate ligament rupture (ROTOR): a randomized controlled clinical trial. BMC Musculoskelet Disord.

[11] Robson AWVI (1903). Ruptured crucial ligaments and their repair by operation. Ann Surg.

[12] van der List JP, DiFelice GS (2017). Primary repair of the anterior cruciate ligament: a paradigm shift. Surgeon.

[13] Taylor SA, Khair MM, Roberts TR (2015). Primary repair of the anterior cruciate ligament: a systematic review. Arthroscopy.

[14] van der List JP, DiFelice GS (2017). successful arthroscopic primary repair of a chronic anterior cruciate ligament tear 11 years following injury. HSS J.

[15] Shah N, Mukhopadhyay R, Vakta R (2018). Suture pullout technique of acute anterior cruciate ligament femoral avulsion repair. Arthrosc Tech.

[16] Praz C, Kandhari VK, Saithna A (2019). ACL rupture in the immediate build-up to the olympic games: return to elite alpine ski competition 5 months after injury and ACL repair. BMJ Case Rep.

[17] van der List JP, DiFelice GS (2017). Range of motion and complications following primary repair versus reconstruction of the anterior cruciate ligament. Knee.

[18] van der List JP, DiFelice GS (2017). Arthroscopic primary anterior cruciate ligament repair with suture augmentation. Arthrosc Tech.

[19] DiFelice GS, Villegas C, Taylor S (2015). Anterior cruciate ligament preservation: early results of a novel arthroscopic technique for suture anchor primary anterior cruciate ligament repair. Arthroscopy.

[20] Bali K, Dhillon MS, Vasistha RK (2012). Efficacy of immunohistological methods in detecting functionally viable mechanoreceptors in the remnant stumps of injured anterior cruciate ligaments and its clinical importance. Knee Surg Sports Traumatol Arthrosc.

[21] Iwasa J, Ochi M, Uchio Y (2006). Decrease in anterior knee laxity by electrical stimulation of normal and reconstructed anterior cruciate ligaments. J Bone Joint Surg Br.

[22] Achtnich A, Herbst E, Forkel P (2016). Acute proximal anterior cruciate ligament tears: outcomes after arthroscopic suture anchor repair versus anatomic single-bundle reconstruction. Arthroscopy.

[23] Grassi A, Carulli C, Innocenti M (2018). New trends in anterior cruciate ligament reconstruction: a systematic review of national surveys of the last 5 years. Joints.

[24] Ciccotti MC, Secrist E, Tjoumakaris F (2017). Anatomic anterior cruciate ligament reconstruction via independent tunnel drilling: a systematic review of randomized controlled trials comparing patellar tendon and hamstring autografts. Arthroscopy.

[25] Frank CB, Jackson DW (1997). The science of reconstruction of the anterior cruciate ligament. J Bone Joint Surg Am.

[26] Laxdal G, Sernert N, Ejerhed L (2007). A prospective comparison of bone-patellar tendon-bone and hamstring tendon grafts for anterior cruciate ligament reconstruction in male patients. Knee Surg Sports Traumatol Arthrosc.

[27] Hoogeslag RAG, Brouwer RW, Boer BC (2019). Acute anterior cruciate ligament rupture: repair or reconstruction? two-year results of a randomized controlled clinical trial. Am J Sports Med.

[28] Kosters C, Glasbrenner J, Spickermann L (2020). Repair with dynamic intraligamentary stabilization versus primary reconstruction of acute anterior cruciate ligament tears: 2-year results from a prospective randomized study. Am J Sports Med.

[29] Murray MM, Fleming BC, Badger GJ (2020). Bridge-enhanced anterior cruciate ligament repair is not inferior to autograft anterior cruciate ligament reconstruction at 2 years: results of a prospective randomized clinical trial. Am J Sports Med.

[30] Schliemann B, Glasbrenner J, Rosenbaum D (2018). Changes in gait pattern and early functional results after ACL repair are comparable to those of ACL reconstruction. Knee Surg Sports Traumatol Arthrosc.

[31] Buckle C, Wainwright AM (2018). A systematic review of long-term patient reported outcomes for the treatment of anterior cruciate ligament injuries in the skeletally immature. J Child Orthop.

[32] Howick J CI, Glasziou P, Greenhalgh T, Carl Heneghan, Liberati A, Moschetti I, Phillips B, Thornton H, Goddard O, Hodgkinson M. The 2011 Oxford CEBM Levels of Evidence. Oxford Centre for Evidence-Based Medicine. 2011.

[33] Burns PB, Rohrich RJ, Chung KC (2011). The levels of evidence and their role in evidence-based medicine. Plast Reconstr Surg.

[34] Giai Via R, Bosco F, Giustra F (2022). Acute rockwood type III ACJ dislocation: conservative vs surgical approach a systematic review and meta-analysis of current concepts in literature. Injury.

[35] Page MJ, Moher D, Bossuyt PM (2021). PRISMA 2020 explanation and elaboration: updated guidance and exemplars for reporting systematic reviews. BMJ.

[36] Liberati A, Altman DG, Tetzlaff J (2009). The PRISMA statement for reporting systematic reviews and meta-analyses of studies that evaluate health care interventions: explanation and elaboration. PLoS Med.

[37] Bistolfi A, Giustra F, Bosco F (2022). Comparable results between crosslinked polyethylene and conventional ultra-high molecular weight polyethylene implanted in total knee arthroplasty: systematic review and meta-analysis of randomised clinical trials. Knee Surg Sports Traumatol Arthrosc.

[38] Risitano S, Cacciola G, Sabatini L (2022). Restricted kinematic alignment in primary total knee arthroplasty: a systematic review of radiographic and clinical data. J Orthop.

[39] Collins NJ, Misra D, Felson DT (2011). Measures of knee function: international knee documentation committee (IKDC) subjective knee evaluation form, knee injury and osteoarthritis outcome score (KOOS), knee injury and osteoarthritis outcome score physical function short form (KOOS-PS), knee outcome survey activities of daily living scale (KOS-ADL), lysholm knee scoring scale, oxford knee score (OKS), western ontario and mcmaster universities osteoarthritis index (WOMAC), activity rating scale (ARS), and tegner activity score (TAS). Arthritis Care Res (Hoboken).

[40] Reed MD, Van Nostran W (2014). Assessing pain intensity with the visual analog scale: a plea for uniformity. J Clin Pharmacol.

[41] Sporsheim AN, Gifstad T, Lundemo TO (2019). Autologous BPTB ACL reconstruction results in lower failure rates than ACL repair with and without synthetic augmentation at 30 years of follow-up: a prospective randomized study. J Bone Joint Surg Am.

[42] Vermeijden HD, van der List JP, O'Brien R (2020). Patients forget about their operated knee more following arthroscopic primary repair of the anterior cruciate ligament than following reconstruction. Arthroscopy.

[43] Biau DJ, Tournoux C, Katsahian S (2007). ACL reconstruction: a meta-analysis of functional scores. Clin Orthop Relat Res.

[44] Steadman JR, Matheny LM, Briggs KK (2012). Outcomes following healing response in older, active patients: a primary anterior cruciate ligament repair technique. J Knee Surg.

[45] Strand T, Molster A, Hordvik M (2005). Long-term follow-up after primary repair of the anterior cruciate ligament: clinical and radiological evaluation 15–23 years postoperatively. Arch Orthop Trauma Surg.

[46] Papalia R, Torre G, Papalia G (2019). Arthroscopic primary repair of the anterior cruciate ligament in adults: a systematic review. Br Med Bull.

[47] Petersen W, Zantop T (2007). Anatomy of the anterior cruciate ligament with regard to its two bundles. Clin Orthop Relat Res.

[48] Gabriel MT, Wong EK, Woo SL (2004). Distribution of in situ forces in the anterior cruciate ligament in response to rotatory loads. J Orthop Res.

[49] Sakane M, Fox RJ, Woo SL (1997). In situ forces in the anterior cruciate ligament and its bundles in response to anterior tibial loads. J Orthop Res.

[50] Amis AA (2017). Anterolateral knee biomechanics. Knee Surg Sports Traumatol Arthrosc.

[51] Beynnon BD, Johnson RJ, Fleming BC (1997). The effect of functional knee bracing on the anterior cruciate ligament in the weightbearing and nonweightbearing knee. Am J Sports Med.

[52] Matsumoto H, Suda Y, Otani T (2001). Roles of the anterior cruciate ligament and the medial collateral ligament in preventing valgus instability. J Orthop Sci.

[53] Schultz RA, Miller DC, Kerr CS (1984). Mechanoreceptors in human cruciate ligaments. a histological study. J Bone Joint Surg Am.

[54] Xu Y, Liu J, Kramer S (2011). Comparison of in situ forces and knee kinematics in anteromedial and high anteromedial bundle augmentation for partially ruptured anterior cruciate ligament. Am J Sports Med.

[55] Hewett TE, Ford KR, Hoogenboom BJ (2010). Understanding and preventing acl injuries: current biomechanical and epidemiologic considerations—update 2010. N Am J Sports Phys Ther.

[56] Ekdahl M, Wang JH, Ronga M (2008). Graft healing in anterior cruciate ligament reconstruction. Knee Surg Sports Traumatol Arthrosc.

[57] Kawamura S, Ying L, Kim HJ (2005). Macrophages accumulate in the early phase of tendon-bone healing. J Orthop Res.

[58] Rougraff BT, Shelbourne KD (1999). Early histologic appearance of human patellar tendon autografts used for anterior cruciate ligament reconstruction. Knee Surg Sports Traumatol Arthrosc.

[59] Arnoczky SP, Tarvin GB, Marshall JL (1982). Anterior cruciate ligament replacement using patellar tendon. an evaluation of graft revascularization in the dog. J Bone Joint Surg Am.

[60] Amiel D, Kleiner JB, Akeson WH (1986). The natural history of the anterior cruciate ligament autograft of patellar tendon origin. Am J Sports Med.

[61] Jackson DW, Corsetti J, Simon TM (1996). Biologic incorporation of allograft anterior cruciate ligament replacements. Clin Orthop Relat Res.

[62] Zaffagnini S, De Pasquale V, Marchesini Reggiani L (2007). Neoligamentization process of BTPB used for ACL graft: histological evaluation from 6 months to 10 years. Knee.

[63] Rougraff B, Shelbourne KD, Gerth PK (1993). Arthroscopic and histologic analysis of human patellar tendon autografts used for anterior cruciate ligament reconstruction. Am J Sports Med.

[64] Park MJ, Lee MC, Seong SC (2001). A comparative study of the healing of tendon autograft and tendon-bone autograft using patellar tendon in rabbits. Int Orthop.

[65] Grossman MG, ElAttrache NS, Shields CL (2005). Revision anterior cruciate ligament reconstruction: three- to nine-year follow-up. Arthroscopy.

[66] Chen L, Cooley V, Rosenberg T (2003). ACL reconstruction with hamstring tendon. Orthop Clin North Am.

[67] Hospodar SJ, Miller MD (2009). Controversies in ACL reconstruction: bone-patellar tendon-bone anterior cruciate ligament reconstruction remains the gold standard. Sports Med Arthrosc Rev.

[68] McGuire DA, Hendricks SD (2009). Allograft tissue in ACL reconstruction. Sports Med Arthrosc Rev.

[69] Eggli S, Kohlhof H, Zumstein M (2015). Dynamic intraligamentary stabilization: novel technique for preserving the ruptured ACL. Knee Surg Sports Traumatol Arthrosc.

[70] Roth JH, Kennedy JC, Lockstadt H (1985). Polypropylene braid augmented and nonaugmented intraarticular anterior cruciate ligament reconstruction. Am J Sports Med.

[71] DiFelice GS, van der List JP (2016). Arthroscopic primary repair of proximal anterior cruciate ligament tears. Arthrosc Tech.

[72] Murray MM, Flutie BM, Kalish LA (2016). The bridge-enhanced anterior cruciate ligament repair (BEAR) procedure: an early feasibility cohort study. Orthop J Sports Med.

[73] Murray MM, Kalish LA, Fleming BC (2019). Bridge-enhanced anterior cruciate ligament repair: two-year results of a first-in-human study. Orthop J Sports Med.

[74] Chahla J, Nelson T, Dallo I (2020). Anterior cruciate ligament repair versus reconstruction: a kinematic analysis. Knee.

[75] Beynnon BD, Fleming BC, Labovitch R (2002). Chronic anterior cruciate ligament deficiency is associated with increased anterior translation of the tibia during the transition from non-weightbearing to weightbearing. J Orthop Res.

[76] Sherman MF, Lieber L, Bonamo JR (1991). The long-term followup of primary anterior cruciate ligament repair. defining a rationale for augmentation. Am J Sports Med.

[77] Toy BJ, Yeasting RA, Morse DE (1995). Arterial supply to the human anterior cruciate ligament. J Athl Train.

[78] Nguyen DT, Ramwadhdoebe TH, van der Hart CP (2014). Intrinsic healing response of the human anterior cruciate ligament: an histological study of reattached ACL remnants. J Orthop Res.

